# The Rise of Oncoendocrinology: How Modern Cancer Therapies Are Reshaping Endocrine Practice

**DOI:** 10.3390/medsci14030347

**Published:** 2026-06-26

**Authors:** Nidha Shapoo, Noella Boma, Vladimir Gotlieb, Joseph Mattana, Regina Belokovskaya, Alberto Franco

**Affiliations:** Department of Medicine, NYC Health & Hospitals/Metropolitan Hospital, New York Medical College, New York, NY 10029, USA; boman1@nychhc.org (N.B.); gotliebv@nychhc.org (V.G.); mattanaj@nychhc.org (J.M.); belokovr@nychhc.org (R.B.); francoa1@nychhc.org (A.F.)

**Keywords:** oncoendocrinology, immunotherapy, targeted therapies, endocrine toxicities, hyperglycemia, thyroid dysfunction, survivorship, future direction, personalized care

## Abstract

The emergence of immune checkpoint inhibitors, targeted therapies, CAR T-cell therapy, and antibody–drug conjugates has transformed modern oncology, significantly improving survival across a wide range of malignancies. However, these advances have also introduced a growing spectrum of endocrine and metabolic complications, redefining the scope of endocrine practice. Therapy-induced endocrinopathies, including thyroid dysfunction, hypophysitis, adrenal insufficiency, diabetes, pancreatitis, dyslipidemia, gonadal dysfunction, and metabolic syndrome, are recognized as clinically significant and often long-lasting consequences of cancer treatment. Unlike classical endocrine disorders, these conditions frequently present abruptly, display atypical clinical patterns, and require complex multidisciplinary management. This review explores the evolving field of oncoendocrinology, focusing on the mechanisms, clinical manifestations, and management of endocrine toxicities associated with novel cancer therapies. We also discuss the long-term endocrine sequelae of cancer treatment and the growing need for structured survivorship care and endocrine surveillance. In addition, we discuss future directions in oncoendocrinology, including predictive biomarkers, the need for treatment standardization, integrated care models, survivorship surveillance, and precision-based approaches to endocrine care. As cancer survival continues to improve, early recognition and long-term management of endocrine complications are becoming essential to optimizing both quality of life and overall outcomes in cancer survivors.

## 1. Introduction

The past two decades have seen an extraordinary transformation in cancer treatment, driven by the introduction of cancer immunotherapy, targeted therapy, chimeric antigen receptor (CAR) T-cell therapy, and antibody–drug conjugates (ADCs) [1,2]. Immunotherapies, such as immune checkpoint inhibitors (ICIs), enhance the immune system’s ability to recognize and attack cancer cells, while targeted therapies inhibit specific molecular pathways that drive tumor growth. These innovations have led to significant improvements in survival across a wide range of malignancies, including melanoma, lung cancer, renal cell carcinoma, and hematologic cancers. Yet these therapeutic advances bring new clinical challenges. Unlike traditional cytotoxic chemotherapy, which is associated with predictable and often reversible toxicities, modern cancer therapies can trigger immune-mediated and metabolic disturbances that affect multiple organ systems [3,4,5,6,7]. Among these, endocrine complications have emerged as the most clinically significant adverse effects. These disorders are not merely side effects; they represent new disease entities that arise directly from the mechanisms that make these therapies effective [8,9,10] (Table 1).

The endocrine system, with its finely tuned regulatory networks, is particularly vulnerable to disruptions in immune tolerance and intracellular signaling. The field of oncoendocrinology has thus emerged as a critical interface between oncology, endocrinology, and immunology, driven primarily by the explosive growth of ICIs in modern cancer therapy [11].

This review examines how modern cancer therapies are reshaping endocrine practice. It explores the mechanisms behind therapy-induced endocrinopathies, the clinical patterns that define these conditions, and the challenges they pose for diagnosis and management. In doing so, it highlights the need for a paradigm shift in how endocrine disorders are conceptualized and treated within cancer care.

## 2. Methods

This narrative review summarizes the current understanding of endocrine and metabolic complications associated with modern cancer therapies, including immune checkpoint inhibitors, targeted therapies, CAR T-cell therapy, and antibody–drug conjugates. A comprehensive literature search was performed using PubMed, MEDLINE, and Google Scholar for articles published in English through 2026. Search terms included combinations of “oncoendocrinology,” “immune checkpoint inhibitors,” “endocrine toxicities,” “immune-related adverse events,” “targeted therapy,” “tyrosine kinase inhibitors,” “PI3K inhibitors,” “mTOR inhibitors,” “CAR T-cell therapy,” “antibody-drug conjugates,” “survivorship endocrinology,” “therapy-induced diabetes,” “thyroid dysfunction,” and “hypophysitis.”

Priority was given to clinical trials, meta-analyses, prospective cohort studies, consensus guidelines, landmark studies, and recent review articles relevant to endocrine complications of cancer therapies. Additional references were identified through manual review of the bibliographies of selected articles and of professional society guidelines, including recommendations from the American Diabetes Association, the National Comprehensive Cancer Network, the European Society of Endocrinology, and the American Society of Clinical Oncology. Articles were selected based on clinical relevance, scientific impact, and contribution to emerging concepts in oncoendocrinology, including survivorship care, risk stratification, and precision medicine.

## 3. The Evolution of Oncoendocrinology

The term oncoendocrinology was first used in the context of hormonal carcinogenesis research examining how endocrine signaling drives hormone-dependent cancers, such as breast and prostate cancers, and how hormonal therapies can be leveraged for treatment [12]. More recently, Takahashi proposed the broader concept of Onco-Immuno-Endocrinology after discovering paraneoplastic autoimmune hypophysitis, a condition in which tumors ectopically express pituitary antigens, thereby breaking immune tolerance and triggering autoimmune destruction of the pituitary. This phenomenon requires a simultaneous understanding of all three disciplines [11].

The introduction of immune checkpoint inhibitors and targeted therapies has dramatically altered this landscape. The most powerful driver of oncoendocrinology’s emergence has been the widespread adoption of ICIs. Endocrine toxicities are now among the most frequently reported adverse events with these treatments. Unlike traditional endocrine disorders, which often develop gradually and are mediated by well-characterized autoimmune or degenerative processes, therapy-induced endocrinopathies can occur abruptly and without warning. They may present with nonspecific symptoms that overlap with those of cancer itself, such as fatigue, weight changes, and weakness, making diagnosis particularly challenging. Routine monitoring of endocrine parameters, such as thyroid function tests, cortisol levels, and glucose levels, has therefore become an essential component of care for patients receiving modern cancer therapies [11]. Moreover, these conditions are often irreversible. While some immune-related adverse events affecting other organ systems may resolve with immunosuppressive therapy, endocrine glands damaged by immune-mediated processes frequently fail to recover. This necessitates lifelong hormone replacement and ongoing monitoring, effectively converting an acute complication into a chronic condition [13].

The field of oncoendocrinology has become increasingly formalized through initiatives such as the EndoCompass Project, a collaborative effort between the European Society of Endocrinology and the European Society for Paediatric Endocrinology that has defined key research priorities shaping the field’s scope. These priorities include the study of endocrine drivers of carcinogenesis, such as the roles of steroid hormone signaling and genomic instability in breast and prostate cancers; the endocrine consequences of cancer therapies and the metabolic complications affecting a substantial proportion of cancer survivors; and paraneoplastic endocrine syndromes, in which ectopic hormone production by tumors can mimic metastatic disease and complicate diagnosis and prognosis [14,15,16].

In parallel, the growing population of long-term cancer survivors has drawn attention to survivorship endocrinology, marked by late effects such as premature menopause, growth hormone deficiency, and metabolic syndrome [17].

These growing clinical demands have spurred the creation of dedicated oncoendocrinology teams within cancer centers, underscoring the need for close collaboration between oncologists and endocrinologists. Multidisciplinary models have been shown to improve outcomes, particularly in managing immune-related endocrine toxicities, which are often permanent and require lifelong follow-up [13]. Supporting this, recent prospective DIRECT cohort data have identified younger age, female sex, and obesity as independent predictors of clinically significant immune checkpoint inhibitor-induced endocrinopathies, underscoring the importance of risk stratification as the field evolves [18].

Collectively, these advances highlight that modern cancer therapies, especially immune checkpoint inhibitors, have made endocrine complications both common and clinically significant, thereby establishing oncoendocrinology as a distinct and essential discipline at the intersection of oncology, endocrinology, and immunology.

## 4. Hormones in Cancer Biology

Endocrine signaling is fundamental to the development and progression of several major malignancies, particularly hormone-sensitive cancers such as breast, prostate, endometrial, and ovarian cancers, which together account for nearly one-third of all cancer cases worldwide [19]. These tumors are strongly influenced by steroid hormones, including estrogens, androgens, and progesterone, which regulate cellular proliferation, differentiation, apoptosis, and genomic stability through complex receptor-mediated signaling pathways. Dysregulation of these pathways can drive oncogenesis by promoting sustained cell growth, inhibiting programmed cell death, and facilitating the accumulation of genetic mutations [14,20].

Beyond classical steroid hormone signaling, metabolic endocrine pathways also play a significant role in tumor biology. The insulin and insulin-like growth factor (IGF) axis has emerged as a critical mediator linking metabolic disorders, such as obesity and type 2 diabetes, to increased cancer risk and progression. Hyperinsulinemia and elevated IGF-1 levels enhance mitogenic signaling, inhibit apoptosis, and promote angiogenesis, creating a favorable environment for tumor growth. This interplay underscores the growing recognition of the relationship between metabolic health and cancer outcomes [21].

In addition to responding to systemic hormonal signals, tumors can actively modulate the endocrine environment. Many cancers produce neurohormonal mediators and cytokines that disrupt normal hypothalamic–pituitary–end-organ axes, effectively “reprogramming” host physiology to support tumor survival and expansion. This bidirectional interaction between tumors and the endocrine system underscores that cancer is not only a localized disease but also a systemic one with widespread hormonal implications [22].

These biological insights underpin endocrine-based therapeutic strategies, including aromatase inhibitors, selective estrogen receptor modulators, anti-androgens, and gonadotropin-releasing hormone agonists, which remain central to the management of hormone-driven malignancies [23]. However, by altering key hormonal pathways, these treatments also introduce a range of downstream endocrine complications, including metabolic disturbances, bone loss, and gonadal dysfunction. Thus, the same pathways targeted for therapeutic benefit also contribute to the expanding field of oncoendocrinology, where understanding the balance between cancer control and endocrine homeostasis is essential.

## 5. Immune Checkpoint Inhibitor-Associated Endocrinopathies

Immune checkpoint inhibitors (ICIs) have revolutionized cancer treatment by enhancing antitumor immunity by inhibiting key regulatory pathways, including cytotoxic T-lymphocyte-associated antigen 4 (CTLA-4) and programmed death receptor-1 (PD-1) or its ligand (PD-L1). By blocking inhibitory signals that normally maintain immune homeostasis, these agents amplify T-cell-mediated immune responses against tumor cells. However, this immune activation is inherently nonspecific and may lead to a breakdown of self-tolerance, resulting in immune-related adverse events (irAEs) that can affect virtually any organ system. Endocrinopathies are among the most common irAEs, affecting up to 40% of treated patients depending on the regimen. These endocrine toxicities are particularly significant because of their frequency, potential severity, and often irreversible nature. As such, they often require lifelong hormone replacement and long-term follow-up. Adverse effects have been reported more frequently with CTLA-4 inhibitors (e.g., ipilimumab) vs. PD-1 inhibitors (e.g., pembrolizumab, nivolumab); however, combination therapy carries a greater risk [15,24].

The spectrum of endocrine complications associated with ICIs is broad, with thyroid dysfunction, hypophysitis, adrenal insufficiency, insulin-dependent diabetes mellitus, and pancreatitis being the most clinically relevant entities [15,25,26,27]. These conditions are characterized by abrupt onset, atypical presentations, and significant overlap with malignant symptoms, posing unique diagnostic and therapeutic challenges.

### 5.1. Thyroid Dysfunction

Thyroid dysfunction is the most frequently encountered endocrine complication of ICIs, with incidence rates varying by agent class and combination regimens. PD-1 and PD-L1 inhibitors are particularly associated with thyroid abnormalities (~10–20%), although the risk is further amplified when combined with CTLA-4 inhibitors (>20%) [15,23]. A large real-world study of 39,749 ICI-treated patients found 14.2% developed hypothyroidism within 12 months, with 72.9% requiring levothyroxine initiation [28,29].

ICI-associated thyroid dysfunction most commonly follows a characteristic clinical course of painless destructive thyroiditis, presenting with transient thyrotoxicosis that progresses to hypothyroidism. The thyrotoxic phase typically develops within 4–8 weeks of therapy initiation, although it may occur as early as 2 weeks with combination immunotherapy, and generally lasts 3–6 weeks, often remaining mild or asymptomatic. This is followed by the onset of hypothyroidism, usually occurring between 10 and 20 weeks after initiation of therapy and frequently representing a permanent state requiring lifelong hormone replacement. Although uncommon, Graves’ disease should be considered in cases of persistent thyrotoxicosis [25,28].

Several risk factors have been identified, including female sex, younger age, higher baseline thyroid-stimulating hormone levels, and pre-existing thyroid autoantibodies. Notably, patients with positive anti-thyroid antibodies have a substantially higher risk of thyroid dysfunction compared with antibody-negative individuals, even in the setting of PD-1 monotherapy. Combination immune checkpoint inhibitor therapy further amplifies this risk, with significantly higher rates of overt thyrotoxicosis observed. Certain malignancies, including renal, endometrial, and hepatic cancers, have also been associated with increased susceptibility [30].

Management of ICI-induced hypothyroidism is often complicated by suboptimal levothyroxine dosing; studies have shown that a significant proportion of patients do not receive appropriate weight-based dosing, leading to delayed normalization of thyroid function. Additionally, patients who experience a preceding thyrotoxic phase may require higher levothyroxine doses, and more rapid titration strategies have been associated with faster achievement of euthyroidism without increased adverse effects [31].

Development of overt thyrotoxicosis has been linked to improved oncologic outcomes, including longer progression-free and overall survival, suggesting that thyroid irAEs may serve as a biomarker of ICI efficacy [31].

### 5.2. Hypophysitis

ICI-induced hypophysitis is one of the common endocrine irAEs. Hypophysitis is most strongly associated with CTLA-4 inhibitors, particularly ipilimumab (14–16%), although it may also occur with PD-1 and PD-L1 inhibitors (0.5%). Combination anti-CTLA-4/PD-1 therapy confers the highest overall incidence, with rates reaching approximately 9–17% [32]. This condition involves immune-mediated inflammation of the pituitary gland, leading to varying degrees of anterior pituitary hormone deficiency.

The pathophysiology of ICI-induced hypophysitis is thought to involve direct immune and complement-mediated targeting of pituitary antigens, potentially facilitated by CTLA-4 expression within the gland [3]. CTLA-4 inhibitors preferentially cause pituitary inflammation with gland enlargement, whereas anti-PD-1/PD-L1-associated hypophysitis tends to present as isolated ACTH deficiency without pituitary enlargement on MRI [33]. In combination immunotherapy, two patterns emerge: combined pituitary hormone deficiency occurring earlier (median 40 days) with headache and MRI changes, versus isolated ACTH deficiency occurring later (median 84 days), suggesting distinct immunopathologic mechanisms [34].

Emerging evidence supports a role for anti-pituitary antibodies (APA) and anti-hypothalamus antibodies (AHA) in predicting ICI-induced pituitary dysfunction. Kobayashi et al. found that APAs were present at baseline in 64.7% of patients who subsequently developed ICI-induced isolated ACTH deficiency, compared with 2.5% of controls (*p* < 0.001). In patients who developed ICI-induced hypophysitis, APAs were negative at baseline but seroconverted before clinical onset. Susceptible HLA alleles (HLA-Cw12, HLA-DR15) were also enriched, suggesting that combined APA and HLA typing could serve as a predictive panel [35].

Bellastella et al. examined APA and AHA in patients treated with anti-PD-1/PD-L1 antibodies and found a significantly higher prevalence of both APA and AHA than in healthy controls. In the longitudinal arm, 53.8% of initially APA-negative patients seroconverted after nine weeks of therapy, accompanied by hormonal changes (rising prolactin, declining ACTH and IGF-1), suggesting that seroconversion may precede clinically overt disease [36]. Additional autoantibody targets—including anti-guanine nucleotide-binding protein G(olf) subunit alpha (anti-GNAL) and anti-integral membrane protein 2B (anti-ITM2B) antibodies, have also been correlated with subsequent hypophysitis [37]. Labadzhyan et al. prospectively confirmed that endocrine-specific autoantibodies were significantly associated with the development of endocrine irAEs [38].

These data suggest that screening for APA and AHA before ICI initiation could identify patients at higher risk, and that serial monitoring of seronegative patients may detect preclinical seroconversion, potentially enabling timely intervention. However, standardized assays are not yet widely available, and consensus guidelines do not yet recommend routine APA/AHA screening. Validation in larger prospective cohorts is needed before clinical implementation.

Clinically, hypophysitis presents with acute or subacute symptoms including fatigue, headache, dizziness, nausea, anorexia, confusion, hypotension due to secondary adrenal insufficiency, and, in severe cases, adrenal crises. These symptoms can be easily attributed to underlying malignancy or systemic therapy, which may delay diagnosis. Some patients present with symptoms from the mass effect of pituitary enlargement (e.g., headache, vision change), more often with anti-CTLA-4 therapies. Central hypothyroidism and hypogonadism may also occur, although these are generally less acutely life-threatening [39].

The development of ICI-induced hypophysitis may be associated with improved oncologic outcomes. A meta-analysis of 43 studies found that endocrine irAEs were associated with significantly improved survival [40]. However, these findings are not uniform, as some analyses lose significance after landmark adjustment for lead-time bias [41]. Notably, high-dose glucocorticoid use for hypophysitis management may negatively impact oncologic outcomes, supporting physiologic replacement doses rather than immunosuppressive dosing [42].

A key feature distinguishing ICI-induced hypophysitis from other forms is the low likelihood of recovery of pituitary function. ACTH deficiency is usually permanent, necessitating lifelong glucocorticoid replacement [39]. TSH and gonadal axis deficits may recover in a substantial proportion of patients, warranting periodic reassessment [43]. When multiple pituitary hormones are deficient, glucocorticoid replacement must precede thyroid hormone replacement to avoid precipitating adrenal crisis [39,43]. Immunotherapy can generally be resumed once acute symptoms resolve and hormone replacement is initiated; permanent discontinuation is not always required.

### 5.3. Adrenal Insufficiency

Adrenal insufficiency (AI) in the context of immune checkpoint inhibition may present as either a primary disorder (high ACTH with low morning cortisol and an abnormal cosyntropin stimulation test) or a secondary disorder (low ACTH with low cortisol), each with distinct pathophysiological mechanisms and clinical implications. Secondary AI is more common and results primarily from ICI-induced hypophysitis [3,32]. Primary AI is rare and results from ICI-induced autoimmune adrenalitis, which predominantly occurs with PD-1 inhibitor monotherapy and often coexists with other endocrinopathies. This form is characterized by deficiencies in both glucocorticoids and mineralocorticoids. Clinically, patients may present with fatigue, hypotension, hyponatremia, hyperkalemia, and, in some cases, hyperpigmentation due to elevated ACTH levels [44].

Distinguishing primary from secondary AI is critically important as primary AI requires both mineralocorticoid and glucocorticoid replacement, whereas secondary AI requires only glucocorticoid replacement. Diagnostic evaluation typically involves measurement of morning cortisol and ACTH levels, along with dynamic testing when necessary [44].

Anti-21-hydroxylase (anti-CYP21) antibodies may serve as predictive biomarkers for ICI-induced primary adrenal insufficiency. These antibodies are detected in ~90% of patients with autoimmune Addison’s disease, typically preceding clinical disease by months to years, and in 30–48% of antibody-positive individuals, overt adrenal insufficiency develops [45]. In the ICI setting, anti-21-hydroxylase antibody positivity has been documented in ICI-induced PAI, confirming an autoimmune etiology [46]. Labadzhyan et al. prospectively demonstrated that endocrine-specific autoantibodies (including adrenal antibodies) were significantly associated with the development of endocrine irAEs [38].

Screening for anti-21-hydroxylase antibodies before ICI initiation could identify patients with subclinical adrenal autoimmunity at heightened risk, and serial monitoring in seronegative patients may detect preclinical seroconversion, enabling timely intervention. However, ICI-induced PAI remains rare (<2%), and current guidelines do not recommend routine screening. Prospective validation in larger cohorts is needed.

Regardless of etiology, adrenal insufficiency represents a potentially life-threatening condition that requires prompt recognition and treatment. Given the nonspecific nature of symptoms and their overlap with other conditions, a high index of suspicion is essential in patients receiving ICIs.

### 5.4. Immune Checkpoint Inhibitor-Associated Diabetes Mellitus

Immune checkpoint inhibitor-associated diabetes mellitus (ICI-DM) is a rare but life-threatening irAE occurring in approximately 0.6–1.4% of patients treated with ICIs. The presentation is often fulminant with a high proportion of patients presenting with diabetic ketoacidosis (DKA) [46].

The pathophysiology of ICI-DM involves rapid immune-mediated destruction of pancreatic beta cells, leading to absolute insulin deficiency. ICI-DM is most strongly associated with anti-PD-1 (nivolumab, pembrolizumab) and anti-PD-L1 (durvalumab, atezolizumab, avelumab) agents. PD-L1 is normally expressed on pancreatic β-cells and serves a protective role. PD-1/PD-L1 blockade disrupts pancreatic immune tolerance, leading to autoreactive CD8+ T-cell-mediated destruction of β cells. Additional contributing factors include genetic susceptibility, particularly high-risk HLA haplotypes identified in a substantial proportion of cases; pre-existing islet autoantibodies (most commonly anti-GAD); cytokine-driven immune activation; and potential perturbations in the incretin axis [47,48].

ICI-DM represents a distinct clinical entity from classical type 1 diabetes, characterized by a lower prevalence of autoantibodies, absence of a typical honeymoon phase, and a more fulminant onset occurring over days to weeks [49].

Clinically, patients often present with severe hyperglycemia, with approximately 70% developing diabetic ketoacidosis at initial presentation. Blood glucose levels are markedly elevated, frequently exceeding 600 mg/dL, while glycated hemoglobin levels are only modestly elevated, reflecting the rapid onset of disease. C-peptide levels are typically low or undetectable, indicating profound insulin deficiency. The median time to onset is approximately 12 weeks following initiation of immune checkpoint inhibitor therapy, although cases have been reported as early as one week and as late as several months after treatment discontinuation [48,49].

Concurrent endocrine irAEs, particularly thyroid dysfunction, are observed in a significant proportion of patients, and most cases require hospitalization at presentation. Given the abrupt onset and severity, current guidelines recommend routine glucose monitoring prior to and during therapy, along with patient education regarding symptoms of hyperglycemia and diabetic ketoacidosis. When hyperglycemia is detected, a structured diagnostic approach is essential, including measurement of C-peptide levels with concurrent glucose, assessment for diabetic ketoacidosis, and evaluation for associated autoantibodies, although the latter are not required for diagnosis. Glucocorticoids are not recommended, as they do not reverse β-cell destruction and may worsen glycemic control. Importantly, immunotherapy can usually be resumed after stabilization, as diabetes is irreversible and discontinuation does not alter its course. Early use of continuous glucose monitoring may further optimize care, and patients should be counseled about long-term insulin dependence and the need for medical-alert identification [46,50].

The development of ICI-DM does not affect overall survival, and patients achieve high objective response rates to immunotherapy [50,51,52].

### 5.5. Immune Checkpoint Inhibitor-Induced Pancreatitis

ICI-induced pancreatic injury is an uncommon but clinically significant gastrointestinal irAE, with an overall incidence of approximately 0.5–5.7%; symptomatic acute pancreatitis occurs in <2% of patients [53]. Combination ICI therapy confers a significantly higher risk (up to 3.8%) compared with monotherapy [54]. The spectrum ranges from asymptomatic lipase elevation (the most common presentation) to fulminant acute pancreatitis with life-threatening consequences; fatal cases have been reported, with a 14.1% mortality rate in a large FAERS pharmacovigilance analysis [55]. The median time to onset is approximately 59 days, with ipilimumab-based regimens showing the shortest latency (~37.5 days) [55]. Importantly, long-term sequelae, including pancreatic atrophy (44–55%), new-onset diabetes mellitus, and exocrine insufficiency, are increasingly recognized, even after initial recovery [53]. Risk factors for ICI-induced pancreatic injury include elevated baseline serum amylase levels and concurrent irAEs in other organs, suggesting that patients with multi-organ immune activation warrant heightened pancreatic surveillance [54]. NCCN guidelines recommend assessment with contrast-enhanced abdominal CT and evaluation for alternative etiologies (alcohol, biliary disease, IgG4-related disease), with management graded by severity, from observation for asymptomatic enzyme elevation to permanent ICI discontinuation for severe (G4) pancreatitis. ASCO guidelines recommend against routine monitoring of amylase or lipase in asymptomatic patients but emphasize prompt workup when suggestive symptoms arise [56]. Given the potential for irreversible pancreatic damage and delayed endocrine/exocrine insufficiency, careful clinical surveillance throughout and after ICI therapy is essential.

## 6. Targeted Therapies and Metabolic Perturbations

The advent of targeted molecular therapies has introduced a new paradigm in oncology, shifting treatment strategies from nonspecific cytotoxic approaches to precision-based interventions targeting dysregulated signaling pathways in tumor cells. These agents, including tyrosine kinase inhibitors, mammalian target of rapamycin (mTOR) inhibitors, and phosphoinositide 3-kinase (PI3K) inhibitors, have significantly improved outcomes across a range of malignancies. However, the signaling pathways they target are not exclusive to malignant cells; they are also integral to normal metabolic and endocrine function. As a result, targeted therapies frequently induce metabolic and endocrine disturbances that are clinically significant and often underrecognized [10,57].

These therapy-induced perturbations reflect the intricate overlap between oncologic pathways and physiological endocrine regulation. Unlike immune-mediated toxicities, which arise from dysregulated immune activation, the metabolic effects of targeted therapies are often the direct consequence of pathway inhibition. This creates a unique set of challenges, as these adverse effects are closely tied to the mechanism of action of the drug and may persist as long as therapy continues.

### 6.1. Tyrosine Kinase Inhibitors-Associated Endocrinopathies

Tyrosine kinase inhibitors (TKIs) have emerged as effective anticancer agents by targeting pathways involved in cellular proliferation, survival, angiogenesis, and metastasis; however, their use is frequently associated with endocrine and metabolic disturbances that can impact quality of life. TKIs cause endocrine adverse effects in a substantial proportion of patients, with thyroid dysfunction (20–30%), dyslipidemia (~50%), and hyperglycemia (15–40%) being the most common. Adrenal insufficiency and gonadal dysfunction are less common but clinically significant [57].

Hypothyroidism is the most common endocrine adverse effect associated with TKIs, occurring in approximately 20–33% of patients, far exceeding the incidence of hyperthyroidism. The underlying mechanisms are multifactorial and include vascular injury from antiangiogenic effects leading to reduced thyroid capillary density and gland atrophy, alterations in deiodinase activity with increased type 3 and decreased type 1 deiodinase resulting in enhanced thyroid hormone clearance, impaired thyroid hormone transport at the pituitary level, and, in some cases, destructive thyroiditis causing an initial thyrotoxic phase followed by hypothyroidism. A distinctive biochemical pattern is often observed, characterized by elevated thyroid-stimulating hormone and a disproportionate reduction in triiodothyronine relative to thyroxine, reflecting altered peripheral metabolism. The onset of thyroid dysfunction varies by agent: some TKIs, such as selpercatinib, cause rapid changes within weeks, whereas others may lead to delayed dysfunction over months to a year [15]. Most vascular endothelial growth factor receptor-targeting (VEGFR) TKIs induce thyroid abnormalities within the first three months of therapy [58]. Given these dynamics, thyroid function should be assessed at baseline and monitored frequently during the first 6 months of treatment, as changes in free thyroxine often precede changes in thyroid-stimulating hormone. While levothyroxine replacement effectively normalizes thyroid-stimulating hormone levels, restoration of triiodothyronine levels may be incomplete in a subset of patients, raising the possibility of a role for combination therapy in select cases [57].

Hyperglycemia is reported in approximately 15–40% of patients receiving TKIs, with certain agents demonstrating a higher metabolic risk profile. Nilotinib has been strongly associated with glucose dysregulation through mechanisms involving impaired insulin secretion, increased peripheral insulin resistance, and altered skeletal muscle glucose utilization. Studies have demonstrated reduced insulin sensitivity and impaired glucose disposal, despite paradoxically elevated insulin responses following glucose administration, suggesting significant post-receptor signaling defects. Nilotinib has also been linked to an increased incidence of metabolic syndrome and dyslipidemia, further compounding cardiovascular risk [49,59]. In contrast, other TKIs, such as imatinib and dasatinib, have been observed to exert favorable metabolic effects, including improved glycemic control and, in some cases, hypoglycemia. These effects are thought to be mediated by enhanced GLUT4 translocation, increased glucose uptake in skeletal muscle, and improved insulin sensitivity [60]. Emerging data also suggest that imatinib may preserve β-cell function and reduce inflammatory signaling, thereby improving glucose homeostasis. These contrasting metabolic profiles highlight the heterogeneity of TKI effects on glucose metabolism, so fasting glucose and lipid profile should be done at baseline and monthly for the first 6 months [57].

Primary adrenal insufficiency (PAI) is an increasingly recognized complication of TKI therapy, with recent prospective studies suggesting a prevalence ranging from 45% to 77%, although many cases remain subclinical [61]. It typically presents with elevated ACTH levels and an inadequate cortisol response to ACTH stimulation testing, with fatigue as the most common, often nonspecific symptom. The onset is variable and may occur within the first year of therapy or after prolonged exposure, necessitating continued surveillance throughout treatment. Several TKIs, including lenvatinib, vandetanib, cabozantinib, sunitinib, and axitinib, have been strongly associated with this complication [62]. Management with physiologic glucocorticoid replacement, such as cortisone acetate, has been shown to significantly improve symptoms and allow continuation of oncologic therapy. Given the subtle presentation, periodic ACTH stimulation testing every 6–8 months is recommended, particularly in patients with lower baseline cortisol reserve, who appear to be at higher risk for developing PAI during treatment [61].

The impact of TKIs on gonadal function remains less clearly defined but is an area of growing clinical interest. Emerging evidence suggests that these agents may exert variable effects on steroidogenesis and reproductive function. For example, imatinib has been associated with subclinical alterations in adrenal steroid pathways, whereas vandetanib has been linked to increased cortisol levels. Long-term exposure to imatinib may adversely affect ovarian reserve and embryo developmental potential, raising important considerations for fertility preservation in younger patients. Additionally, several TKIs have demonstrated antiandrogenic, estrogenic, and antiestrogenic effects in vitro, indicating potential disruptions in hormonal signaling pathways. Although the clinical significance of these findings remains unclear, they underscore the need for further research and careful endocrine evaluation in patients undergoing long-term targeted therapy [57].

### 6.2. mTOR Inhibitors-Associated Endocrinopathies

mTOR inhibitors, including everolimus, temsirolimus, and sirolimus, play a critical role in treating various malignancies by targeting the mTOR pathway, a central regulator of cell growth, proliferation, and metabolism. However, inhibiting this pathway leads to a spectrum of endocrine and metabolic disturbances, most notably affecting glucose and lipid metabolism and male gonadal function [10].

Hyperglycemia and new-onset diabetes are among the most common complications, with reported incidences of 12% to 50% for all-grade hyperglycemia and up to 22% for severe cases. Clinical trials have shown significant increases in hyperglycemia rates compared with placebo, particularly in patients with metastatic renal cell carcinoma [63]. The underlying mechanism involves impaired insulin secretion from direct effects on pancreatic β-cells and increased insulin resistance from disruption of intracellular insulin signaling pathways. Unlike type 2 diabetes, mTOR inhibitor-associated hyperglycemia is often reversible with dose reduction or discontinuation, although it may develop insidiously over weeks to months [10].

Dyslipidemia is another prominent metabolic effect, with high rates of hypertriglyceridemia and hypercholesterolemia observed in clinical studies, often exceeding 70% in treated patients. These abnormalities are thought to result from decreased lipoprotein lipase activity, which impairs clearance of triglyceride-rich lipoproteins, and from reduced LDL receptor expression, which diminishes LDL catabolism. Lipid abnormalities typically emerge within the first year of therapy and may contribute to increased cardiovascular risk, necessitating routine monitoring and management [63,64].

In addition to metabolic effects, mTOR inhibitors, particularly sirolimus, have been linked to male hypogonadism, marked by reduced testosterone levels, elevated gonadotropins, and impaired spermatogenesis. Experimental and clinical data suggest disruption of steroidogenesis and direct effects on testicular function, though these changes are often reversible after discontinuing therapy [65].

Given the high prevalence of metabolic complications, regular monitoring of glucose and lipid parameters is essential in patients receiving mTOR inhibitors. Current recommendations include baseline and periodic assessment of blood glucose and glycated hemoglobin, along with routine lipid profiling, to enable early detection and timely management of these adverse effects [45].

### 6.3. Phosphatidylinositol 3-Kinase Inhibitor-Induced Hyperglycemia

Phosphatidylinositol 3-kinase (PI3K) and the associated PI3K/AKT signaling pathway have been shown to play pivotal roles in oncogenic processes, including cell survival, proliferation, and metabolism. Their hyperactivation leads to tumor growth and metastasis. Hence, PI3K has become an important target for anticancer treatment, especially in hormone receptor-positive metastatic breast cancer [66].

The PI3K/AKT signaling pathway plays a central role in insulin-mediated glucose uptake and overall metabolic regulation. PI3K inhibitors are predominantly associated with hyperglycemia and drug-induced diabetes. By impairing PI3K signaling, these agents reduce GLUT4 translocation and peripheral glucose uptake, leading to marked insulin resistance. Among these, alpelisib is most strongly associated with hyperglycemia, with reported incidence rates of 60–65% for all-grade events and 1 in 3 patients developing severe hyperglycemia, often within the first 2 weeks of therapy. Similar rates have been observed with newer agents, such as inavolisib, while pan-PI3K inhibitors have demonstrated high rates of transient hyperglycemia. In contrast, isoform-selective inhibitors targeting PI3Kδ or γ are less frequently associated with clinically significant metabolic derangements, underscoring the central role of the α-isoform in glucose homeostasis [10].

Unlike immune-mediated diabetes, PI3K inhibitor-induced hyperglycemia is typically reversible with dose modification or temporary interruption of therapy. However, severe complications such as diabetic ketoacidosis, although less common, have been reported with significantly increased risk compared with other anticancer therapies. Identified risk factors include elevated baseline glycated hemoglobin, higher body mass index, preexisting glucose intolerance, older age, and certain ethnic predispositions [10]. Given the high incidence and early onset of hyperglycemia, the ADA recommends a baseline assessment of glucose and glycated hemoglobin, followed by random plasma glucose measurements weekly for the first 2 weeks, then every 4 weeks during treatment, and glycated hemoglobin every 3 months during treatment [46].

Management includes a stepwise approach that avoids insulin when possible. Metformin is started as a first-line agent, followed by pioglitazone or SGLT2 inhibitors in selected patients, while insulin is generally reserved for severe or refractory cases due to concerns regarding potential interference with antitumor efficacy [46].

Dose adjustment or temporary interruption of PI3K inhibitors may be necessary.

## 7. CAR T-Cell Therapy-Associated Endocrinopathies

CAR T-cell therapy is an advanced form of adoptive cellular immunotherapy that has demonstrated remarkable efficacy in relapsed or refractory hematologic malignancies, including diffuse large B-cell lymphoma, acute lymphoblastic leukemia, and multiple myeloma. Despite its transformative impact on cancer outcomes, this therapy is associated with unique toxicities, most notably cytokine release syndrome (CRS) and immune effector cell-associated neurotoxicity syndrome (ICANS). In contrast to immune checkpoint inhibitors, which frequently cause well-characterized endocrine disorders, endocrine complications following CAR T-cell therapy remain underrecognized, less common, and often occur in the context of systemic inflammatory responses such as CRS [7].

The pathophysiology of CAR T-cell-associated endocrinopathies involves a combination of cytokine-driven inflammation, immune dysregulation, and treatment-related factors. The intense inflammatory response associated with CRS can directly impair endocrine organ function, particularly affecting glucose metabolism and adrenal regulation. Additionally, the use of lymphodepleting chemotherapy prior to CAR T-cell infusion and corticosteroids during toxicity management may contribute to endocrine abnormalities, including gonadal dysfunction and secondary adrenal suppression. Unlike checkpoint inhibitor-induced endocrinopathies, which are often autoimmune and irreversible, endocrine effects of CAR T-cell therapy may be transient and closely linked to the acute treatment phase [7].

Hyperglycemia is the most reported endocrine abnormality, with rates approaching 40% among patients with CRS. This is likely driven by the proinflammatory cytokine milieu, particularly interleukin-6, tumor necrosis factor-α, and interferon-γ, which induce insulin resistance and impair glucose metabolism, often compounded using high-dose corticosteroids for CRS or ICANS management [7].

Other reported endocrine abnormalities include adrenal insufficiency, hypothalamic–pituitary dysfunction, and gonadal hormone deficiencies, although these findings are largely derived from pharmacovigilance data and small observational studies. Adrenal dysfunction may result from cytokine-mediated suppression of the hypothalamic–pituitary–adrenal axis or secondary effects of prolonged corticosteroid exposure, while rare cases of central diabetes insipidus suggest possible immune-mediated pituitary injury [67].

Thyroid dysfunction appears to be uncommon in CAR T-cell recipients. Although isolated case reports have described autoimmune thyroiditis following therapy [67], larger cohort analyses have not demonstrated a significant increase in thyroid disease compared with matched controls, suggesting that clinically meaningful thyroid dysfunction is rare at the population level. Similarly, disturbances in calcium homeostasis, such as hypocalcemia, have been reported but remain poorly understood and likely reflect complex interactions between cytokine signaling, renal function, and parathyroid activity.

Recognition of endocrine complications in this setting remains challenging due to the overlap of symptoms with CRS and critical illness. Current guidelines primarily focus on monitoring for metabolic disturbances, such as hyperglycemia, through routine laboratory evaluation, but do not provide specific recommendations for endocrine surveillance. However, emerging evidence suggests that endocrine abnormalities, particularly in conjunction with CRS, may be associated with worse outcomes, underscoring the importance of early identification and management. In practice, close monitoring of glucose levels during the acute phase of therapy, vigilance for symptoms of adrenal insufficiency following corticosteroid taper, and targeted endocrine evaluation in symptomatic patients are essential. As the use of CAR T-cell therapy expands, further research is needed to better characterize the incidence, mechanisms, and long-term consequences of these endocrine effects, and to establish evidence-based guidelines for screening and management [7].

## 8. Antibody–Drug Conjugates-Induced Hyperglycemia

Antibody–drug conjugates (ADCs) are a class of targeted anticancer therapies that combine a monoclonal antibody directed against tumor-specific antigens with a cytotoxic payload, enabling selective delivery of chemotherapy to malignant cells while minimizing systemic toxicity. Although ADCs have significantly advanced cancer treatment, their intrinsic endocrine toxicity profile is relatively limited compared with other modern therapies [68].

The primary endocrinopathy directly attributable to ADCs is hyperglycemia, most notably associated with agents such as enfortumab vedotin and brentuximab vedotin, both of which utilize the monomethyl auristatin E (MMAE) payload. Enfortumab vedotin has been linked to clinically significant hyperglycemia, including severe cases and DKA, with reported incidences of up to 17% for all-grade events and approximately 7% for severe hyperglycemia [69,70]. Similar, though less frequent, effects have been observed with brentuximab vedotin.

The underlying mechanism remains incompletely understood but is thought to involve cytotoxic stress and exacerbation of insulin resistance related to the MMAE payload. Identified risk factors include elevated body mass index, higher baseline glycated hemoglobin, and pre-existing diabetes. Importantly, other endocrine toxicities such as thyroid dysfunction, hypophysitis, and adrenal insufficiency are not intrinsic to ADCs and are more commonly attributable to concurrent ICI therapy, which is frequently used in combination regimens [10].

The critical clinical distinction when hyperglycemia develops in a patient receiving an ADC plus ICI is determining whether it reflects ADC-mediated insulin resistance (appropriate C-peptide) versus ICI-DM (low C-peptide), as management differs substantially; the latter requires lifelong insulin and carries risk of DKA [10].

These findings highlight that, while endocrine complications with ADCs are relatively uncommon, hyperglycemia remains an important and potentially serious adverse effect requiring clinical vigilance.

## 9. Endocrine Monitoring on Modern Cancer Therapies

Given the increasing prevalence and often irreversible nature of therapy-induced endocrinopathies, proactive endocrine monitoring has become an essential component of modern cancer care. Baseline assessment prior to initiation of therapy should include evaluation of thyroid function, glucose metabolism, adrenal function when clinically indicated, and metabolic risk factors. The frequency and intensity of monitoring should be individualized based on the specific anticancer therapy, underlying comorbidities, and the patient’s risk profile. Immune checkpoint inhibitors require regular surveillance for thyroid dysfunction, adrenal insufficiency, and autoimmune diabetes, while therapies targeting the PI3K/AKT/mTOR pathway necessitate close metabolic monitoring due to the high risk of hyperglycemia and dyslipidemia. In addition to laboratory assessment, clinicians should maintain a high index of suspicion for nonspecific symptoms such as fatigue, weight changes, hypotension, or polyuria, which may represent early endocrine toxicity [10,24,26,27,46,56,66] (Table 2).

**Figure 1:**
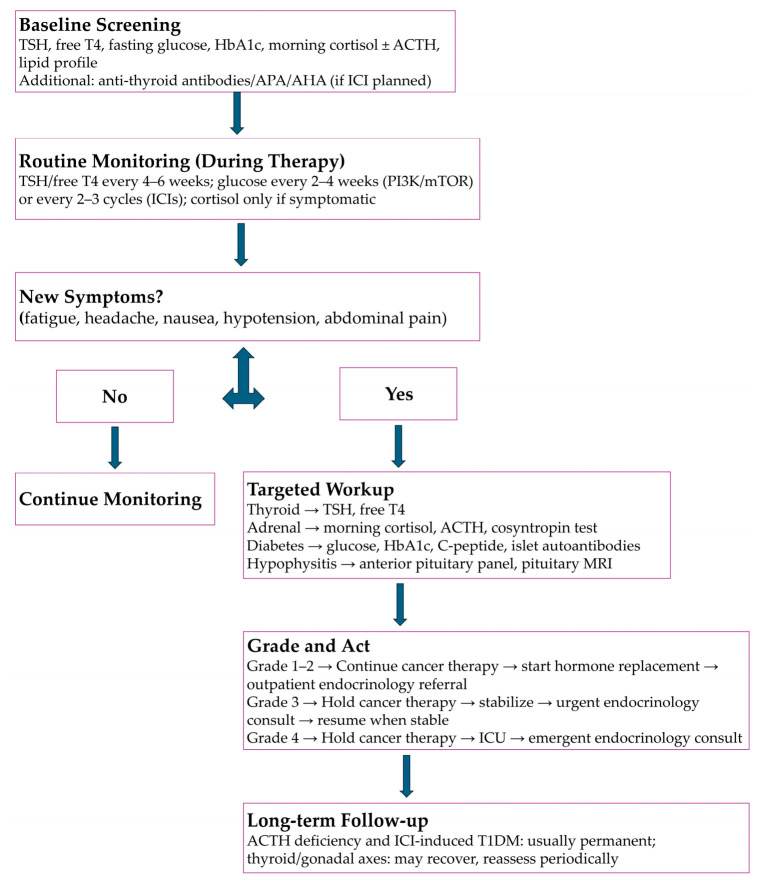
Algorithm for Management of Endocrine Complications During Cancer Therapy.

To operationalize these monitoring principles, we propose a stepwise diagnostic and management algorithm (Figure 1) that integrates baseline screening, symptom-triggered evaluation, severity grading, and cancer therapy continuation decisions for endocrine and metabolic complications across modern cancer therapies.

## 10. Prognostic Significance of Endocrine irAEs by Organ System

The prognostic significance of endocrine irAEs appears to differ by organ system. Thyroid irAEs have the most consistent association with improved survival [31]. A prospective study in NSCLC confirmed that destructive thyroiditis was associated with longer OS, while isolated hypothyroidism without prior thyrotoxicosis was not [71]. In a multicenter retrospective study, endocrine-predominant irAE clusters were associated with favorable survival, comparable to that of cutaneous irAE clusters, whereas respiratory and neurological clusters were associated with unfavorable outcomes [72]. A meta-analysis in hepatocellular carcinoma similarly found that dermatologic and endocrine irAEs were associated with improved survival, while pulmonary, gastrointestinal, and hepatobiliary irAEs were not [73]. Kotwal et al. noted that ICI-thyroiditis has been the endocrinopathy most consistently associated with improved survival, while other endocrinopathies have not shown a significant association [13].

These differential prognostic effects may reflect distinct immunopathologic mechanisms. Thyroid irAEs, particularly destructive thyroiditis, appear to involve a broad breach of self-tolerance driven by cytotoxic CD4 T cells activated against thyroid autoantigens such as thyroglobulin, with expansion of central and effector memory CD4 T cell subsets expressing granzyme B [74]. This process may serve as a surrogate marker of systemic immune activation, as PD-1 blockade simultaneously unleashes both antitumor and autoreactive T cells through shared mechanisms of tolerance disruption [75]. Preexisting antithyroid antibodies (TPO, thyroglobulin) are present in 40–50% of patients who develop thyroid irAEs, and their elevation during ICI therapy suggests that PD-1/PD-L1 blockade broadly modulates both cellular and humoral immunity [28]. In contrast, hypophysitis, particularly anti-CTLA-4-associated hypophysitis, is driven by a more organ-specific mechanism involving direct complement-mediated targeting of CTLA-4 expressed on normal pituitary cells, representing a localized autoimmune process rather than a marker of generalized immune activation [3]. This mechanistic distinction may explain why thyroid irAEs more reliably correlate with antitumor efficacy, whereas the prognostic significance of hypophysitis remains less consistent across studies [13].

## 11. Survivorship and Long-Term Endocrine Sequelae

As cancer survival continues to improve, the long-term endocrine consequences of cancer therapy are becoming an increasingly critical component of survivorship care. Endocrine disorders are particularly prevalent among childhood cancer survivors, affecting up to half of this population, but are now increasingly recognized in adult cancer survivors as well [75].

Gonadal dysfunction is among the most common late effects, resulting from exposure to alkylating agents, pelvic or gonadal radiation, and hormonal therapies, all of which may lead to premature ovarian insufficiency, impaired spermatogenesis, or Leydig cell dysfunction. To facilitate early detection, risk-stratification models based on cumulative treatment exposure have been developed to guide surveillance strategies. Bone health is also profoundly affected by cancer therapies, with aromatase inhibitors, androgen deprivation therapy, glucocorticoids, and chemotherapy-induced premature menopause contributing to accelerated bone loss and increased fracture risk. In breast cancer survivors, treatment-related bone loss is highly prevalent, while androgen deprivation therapy in prostate cancer is associated not only with osteoporosis but also with metabolic complications, including insulin resistance, dyslipidemia, and increased cardiovascular risk. Hypothalamic–pituitary dysfunction is another important late effect, particularly after cranial irradiation, where higher radiation doses are associated with growth hormone deficiency, gonadotropin deficiency, and central adrenal insufficiency. Thyroid dysfunction is similarly common after neck irradiation, with survivors demonstrating substantially increased risks of hypothyroidism and secondary thyroid malignancies. In addition, metabolic syndrome has emerged as a major survivorship concern, driven by the combined effects of radiation exposure, corticosteroids, hormonal therapies, and altered body composition, ultimately contributing to long-term cardiovascular morbidity [15,75,76]. These complications underscore the growing need for structured endocrine surveillance and multidisciplinary survivorship care in patients treated for cancer.

## 12. Future Directions

The field of oncoendocrinology is rapidly evolving, shaped by the accelerating pace of innovation in cancer therapeutics and the growing recognition of endocrine complications as a significant component of treatment-related morbidity. Despite substantial advances in understanding the clinical spectrum of these disorders, many critical questions remain unanswered. Addressing these gaps will be essential not only to improve patient outcomes but also to refine the integration of endocrine care into modern oncology practice (Table 3).

### 12.1. Predictive Biomarkers

One of the most promising areas of investigation is the identification of biomarkers that predict the development of endocrine toxicities. At present, the occurrence of therapy-induced endocrinopathies remains largely unpredictable, with patients often presenting abruptly and without clear predisposing factors. Emerging research has focused on immunologic and genetic markers that may correlate with susceptibility to immune-related adverse events, including endocrine dysfunction. Pre-treatment endocrine-specific autoantibodies (e.g., anti-TPO, anti-GAD) have been shown to be significantly associated with subsequent development of endocrinopathy. Elevated baseline CXCL10 and TSH levels may also predict thyroid irAEs. The nationwide DiRECT cohort identified younger age, female sex, and obesity as independent predictors of grade ≥ 2 endocrinopathies. Derived neutrophil-to-lymphocyte ratio (dNLR ≥ 3) is another emerging predictor. FDG PET/CT may help detect endocrine irAEs before clinical or biochemical manifestation [8,18,38,77]. The successful identification and validation of such biomarkers would represent a major advance, enabling a more personalized approach to cancer therapy in which the risks of endocrine toxicity can be anticipated and mitigated through tailored surveillance and early intervention.

### 12.2. Understanding of Immunopathogenesis

A major unresolved challenge in oncoendocrinology is the incomplete understanding of the mechanisms underlying immune checkpoint inhibitor-associated endocrinopathies. The relative contributions of autoreactive T-cell activation, antibody-mediated injury, and other immune pathways remain unclear, highlighting an important area for ongoing research [25].

### 12.3. Endocrine Toxicity-Tumor Response Correlation

Several studies have demonstrated that the development of endocrine immune-related adverse events, particularly thyroid dysfunction, may be associated with improved progression-free and overall survival in patients receiving immunotherapy, although the underlying mechanisms remain incompletely understood. This observation has led to growing interest in whether endocrine toxicities reflect a more robust antitumor immune response or a distinct immunologic phenotype associated with treatment efficacy. At the same time, an important emerging area of research involves developing strategies to prevent or mitigate endocrine toxicity without compromising oncologic benefit. Unlike many non-endocrine immune-related adverse events, endocrine gland injury is often irreversible, and the role of immunosuppressive therapy in preventing permanent dysfunction remains unclear. Improved understanding of the mechanisms underlying selective involvement of endocrine organs may enable the development of targeted approaches that preserve hormonal function while maintaining effective antitumor immunity. Clarifying the relationship between endocrine toxicities and therapeutic response may ultimately have significant implications for prognostication, risk stratification, and treatment decision-making in patients receiving modern cancer therapies [31,76].

### 12.4. Integrated Care Models and Treatment Standardization

The development of standardized, evidence-based clinical guidelines remains a major priority in oncoendocrinology, as significant variability persists in screening protocols, diagnostic thresholds, and treatment strategies for therapy-induced endocrinopathies. Ongoing controversies include the optimal frequency of biochemical monitoring during immune checkpoint inhibitor therapy, thresholds for initiating hormone replacement in subclinical disease, and management approaches for targeted therapy-induced hyperglycemia. As modern cancer therapies become increasingly complex, endocrine complications must be integrated into routine oncologic care rather than managed as isolated adverse events. This has created a growing need for dedicated oncoendocrinology teams comprising oncologists, endocrinologists, primary care physicians, and other specialists who work through shared care pathways and standardized referral systems. Incorporating endocrine surveillance into oncology workflows, including electronic health record-based alerts and clinical decision-support tools, may facilitate earlier recognition and intervention [78].

### 12.5. Digital Health and Remote Monitoring

Advances in digital health technologies hold promise, particularly for improving the detection and management of therapy-induced endocrine and metabolic complications. Continuous glucose monitoring, remote monitoring platforms, and patient-centered digital tools are emerging as valuable adjuncts for early detection and longitudinal management. Current recommendations emphasize baseline glycemic assessment with fasting glucose and glycated hemoglobin in patients initiating potentially diabetogenic therapies, with selective use of oral glucose tolerance testing in high-risk individuals. The expanding role of continuous glucose monitoring in this setting remains an active area of investigation and may further improve risk stratification and real-time management of therapy-induced metabolic dysfunction [10,79].

### 12.6. Survivorship Endocrinology

As cancer survival continues to improve, the burden of long-term endocrine sequelae is becoming increasingly prominent, positioning survivorship endocrinology as a critical component of cancer care. The EndoCompass roadmap emphasizes the importance of structured surveillance for endocrine complications in cancer survivors, particularly among childhood cancer survivors, as well as the need to better understand treatment-related fertility impairment and familial cancer syndromes with endocrine manifestations [80]. Proposed survivorship frameworks advocate endocrine evaluation before, during, and after cancer therapy, with focused monitoring of thyroid, adrenal, gonadal, and metabolic function. Unlike many treatment-related adverse effects, endocrine dysfunction is frequently permanent and often requires lifelong management [15]. However, the long-term impact of these disorders on quality of life, functional status, cardiovascular health, metabolic outcomes, and psychosocial well-being remains incompletely understood. Persistent questions regarding optimal management of chronic hormone deficiencies, late metabolic complications, and interactions with other comorbidities underscore the need for longitudinal studies and dedicated survivorship cohorts to guide evidence-based long-term care strategies.

### 12.7. Precision Medicine and Personalized Care

Advances in molecular profiling are reshaping the understanding of endocrine tumors, challenging the traditional distinction between endocrine and non-endocrine neoplasms by revealing overlapping oncogenic pathways and shared molecular drivers. These insights are expanding opportunities for targeted and personalized therapeutic strategies [81]. The growing integration of comprehensive tumor genomic profiling, liquid biopsy technologies such as ESR1 mutation detection, and biomarker-guided treatment selection is paving the way for a more precise, individualized approach to oncoendocrinology [82].

## 13. Conclusions

The rapid evolution of modern cancer therapies has improved cancer outcomes but has also introduced a broad spectrum of endocrine and metabolic complications that are increasingly encountered in clinical practice, reshaping the practice of endocrinology. Many of these endocrinopathies are chronic or irreversible, making long-term surveillance and survivorship-focused care essential. Oncoendocrinology has therefore emerged as a critical multidisciplinary field at the intersection of oncology, endocrinology, metabolism, and immunology. As cancer survival continues to improve, there is a growing need for predictive biomarkers, standardized management strategies, integrated care models, and personalized approaches to monitoring and management. Ultimately, the expanding role of the endocrinologist will be central to ensuring that patients not only survive cancer but also maintain long-term endocrine health and quality of life.

## Figures and Tables

**Table 1:** Major Endocrine and Metabolic Complications of Modern Cancer Therapies.

Therapy Class	Mechanism of Action	Major Endocrine/Metabolic Complications
Immune Checkpoint Inhibitors (ICIs)	Enhance T-cell-mediated antitumor immunity through PD-1/PD-L1 and CTLA-4 blockade	Thyroiditis, hypothyroidism, hypophysitis, adrenal insufficiency, autoimmune diabetes
Tyrosine Kinase Inhibitors (TKIs)	Inhibit oncogenic signaling pathways involved in proliferation and angiogenesis	Hypothyroidism, hyperglycemia, dyslipidemia, adrenal dysfunction, gonadal effects
mTOR Inhibitors	Inhibit mTOR signaling involved in cellular growth and metabolism	Hyperglycemia, diabetes, hyperlipidemia, hypogonadism
PI3K Inhibitors	Block PI3K-mediated insulin signaling pathways	Severe hyperglycemia, diabetes, dyslipidemia, diabetic ketoacidosis
CAR T-Cell Therapy	Genetically engineered T cells targeting tumor antigens	CRS-associated hyperglycemia, adrenal dysfunction, pituitary abnormalities
Antibody–Drug Conjugates (ADCs)	Monoclonal antibodies linked to cytotoxic payloads	Hyperglycemia, diabetic ketoacidosis (mainly MMAE-containing ADCs)

**Table 2:** Practical Endocrine Monitoring for Patients Receiving Modern Cancer Therapies.

Therapy Class	Baseline Evaluation	Monitoring During Therapy	Long-Term Monitoring
Immune Checkpoint Inhibitors (ICIs)	TSH, free T4, anti-thyroid antibodies (TPO, thyroglobulin), fasting glucose/HbA1c, morning cortisol ± ACTH	TSH/free T4 every 4–6 weeks, fasting glucose and/or HbA1c every 2–3 cycles; evaluate cortisol if symptomatic	Lifelong monitoring for persistent hypothyroidism, adrenal insufficiency, or diabetes
Tyrosine Kinase Inhibitors (TKIs)	TSH, free T4, fasting glucose/HbA1c, lipid profile ± cortisol, bone density assessment (DEXA), testosterone/estradiol, LH, FSH	Thyroid function every 4–6 weeks during first 6 months; periodic glucose/lipid monitoring	Monitor for chronic hypothyroidism, metabolic syndrome, gonadal dysfunction
mTOR Inhibitors	Fasting glucose/HbA1c, lipid profile	Glucose every 2–4 weeks initially; HbA1c every 3 months; lipid monitoring	Surveillance for diabetes, dyslipidemia, cardiovascular risk
PI3K Inhibitors	Fasting glucose, HbA1c, lipid profile, BMI assessment.	Weekly glucose monitoring during first 2 weeks, then every 4 weeks; HbA1c every 3 months	Long-term metabolic monitoring in high-risk patients
CAR T-Cell Therapy	Baseline glucose, electrolytes, endocrine history	Daily glucose/metabolic monitoring during CRS; assess adrenal function if clinically indicated	Evaluate persistent pituitary dysfunction (cortisol, ACTH, TSH, free T4) if symptomatic.
Antibody–Drug Conjugates (ADCs)	Fasting glucose/HbA1c, diabetes risk assessment	Periodic glucose monitoring, especially during first 4–6 weeks	Monitor for persistent or recurrent hyperglycemia

**Table 3:** Future Directions.

Future Directions in Oncoendocrinology
1. Predictive Biomarkers
2. Understanding of Immunopathogenesis
3. Endocrine Toxicity-Tumor Response Correlation
4. Integrated Care Models and Treatment Standardization
5. Digital Health and Remote Monitoring
6. Survivorship Endocrinology
7. Precision Medicine and Personalized Care

## Data Availability

No new data were created or analyzed in this study.
